# Home environment: respiratory and allergic phenotypes from birth to age six in the PELAGIE cohort

**DOI:** 10.1038/s41533-019-0141-y

**Published:** 2019-07-25

**Authors:** Katharina Apel, Nathalie Costet, Anthony Chapron, Sylvaine Cordier, Christine Monfort, Cécile Chevrier, Fabienne Pelé

**Affiliations:** 1 Univ Rennes, Inserm, EHESP, Irset (Institut de recherche en santé, environnement et travail) - UMR_S 1085, F-35000 Rennes, France; 2 Univ Rennes, Department of General Practice, F-35000, France; 3 Univ Rennes, CHU Rennes, Inserm, CIC 1414 [(Centre d’Investigation Clinique de Rennes)], F-35000 Rennes, France

**Keywords:** Epidemiology, Asthma, Respiratory signs and symptoms, Skin manifestations

## Abstract

Childhood asthma and allergies are particularly prevalent diseases. Our objective is to identify respiratory and allergic phenotypes from birth to 6 years of age, and to explore their environmental determinants, especially those related to the home environment. Data on respiratory and allergic health outcomes and domestic environmental exposure were collected for 935 mother–infant pairs from a longitudinal mother–child cohort based on mothers, included before 19 weeks of gestation in Brittany between 2002 and 2006. Information was obtained by self-administered questionnaires completed by parents at inclusion, delivery, and when the child was 2 and 6 years old. Kml3D clustering was used to describe profiles of children who shared similar trajectories of symptoms as phenotypes. Association with environmental determinants was estimated by polytomous logistic regression. Five phenotypes were identified: a reference group characterized by low symptom levels (31.1%), a transient cough phenotype (36.5%), an eczema/cough phenotype (12.3%), a wheeze/cough phenotype (11.8%), and finally a mixed phenotype (8.0%). The wheeze/cough profile was associated with postnatal exposure to glues used in renovation activities (aOR 2.3 [1.2–4.7]), and the mixed phenotype with postnatal exposure to paint (aOR 2.1 [1–4.5]). The phenotypes observed showed some consistencies with those seen in previous studies. Some exposures associated with respiratory/allergic phenotypes observed in this study are avoidable. If confirmed by further research including interventional trials, home-based environmental counseling could be a possible prevention target for primary care professionals.

## Introduction

The prevalence of childhood asthma and allergies varies greatly worldwide:^1^ at age 6–7, 2.8–37.6% of children have had asthma symptoms in the past year and 2–22.3% eczema symptoms. Asthma is the most frequent chronic disease in childhood and represents a major burden for patients, their families, and society.^2^

Asthma and allergic disorders commonly appear in infancy, but their pathogenesis and interplay remain incompletely understood.^3^ To date, only three studies have collected longitudinal data for multiple 12-month profiles of respiratory and allergic symptoms.^4–6^ Rancière et al.^4^ simultaneously observed profiles of wheezing, dry night cough, eczema, and rhinitis symptoms from ages 0 to 4 in 2522 children from the PARIS (Pollution and Asthma Risk: an Infant Study) birth cohort. Two transient (rhinitis or wheeze) and two persistent (cough/rhinitis or dermatitis) phenotypes were identified, with a low symptoms reference group. Panico et al.^6^ analyzed wheezing and proxies for atopy (eczema and/or hay fever ever) in 11,632 children from the Millennium Cohort Study at ages 3, 5, and 7 and obtained a 4-trajectory clustering: a group with low symptom levels, another with low wheeze and high atopic symptoms, a third with high levels of both wheeze and atopic symptoms, and finally a group with high levels of wheeze only. Belgrave et al.^5^ studied wheeze, eczema, and rhinitis at ages 1, 3, 5, 7, and 11 in 9801 children from the ALSPAC (The Avon Longitudinal Study of Parents and Children) and MAAS (Manchester Asthma and Allergy Study) birth cohorts and identified eight developmental profiles: no disease, atopic march, persistent eczema and wheeze, persistent eczema with later-onset rhinitis, persistent wheeze with later-onset rhinitis, transient wheeze, eczema only, and rhinitis only.

The development and expression of asthma/allergic disorders depend on gene–environment interactions.^7^ Worldwide variations in prevalence rates of asthma and allergies call attention to the potential role of the environment, as genetic differences alone cannot explain this heterogeneity.^1^ The literature has identified numerous avoidable determinants for respiratory and allergic disorders in childhood, in particular, the indoor environment,^8–10^ because young children usually spend most of their time at home.^9,11,12^ The objective of this study was to describe the joint trajectory of asthma, eczema, and rhinitis symptoms at three time points, at ages 1, 2, and 6 years, in a population-based cohort conducted in Brittany (France) from 2002 to 2006. We explored the associations of these phenotypes with some avoidable indoor environmental factors in early life.

## Results

### Participants and descriptive data

**Fig. 1:**
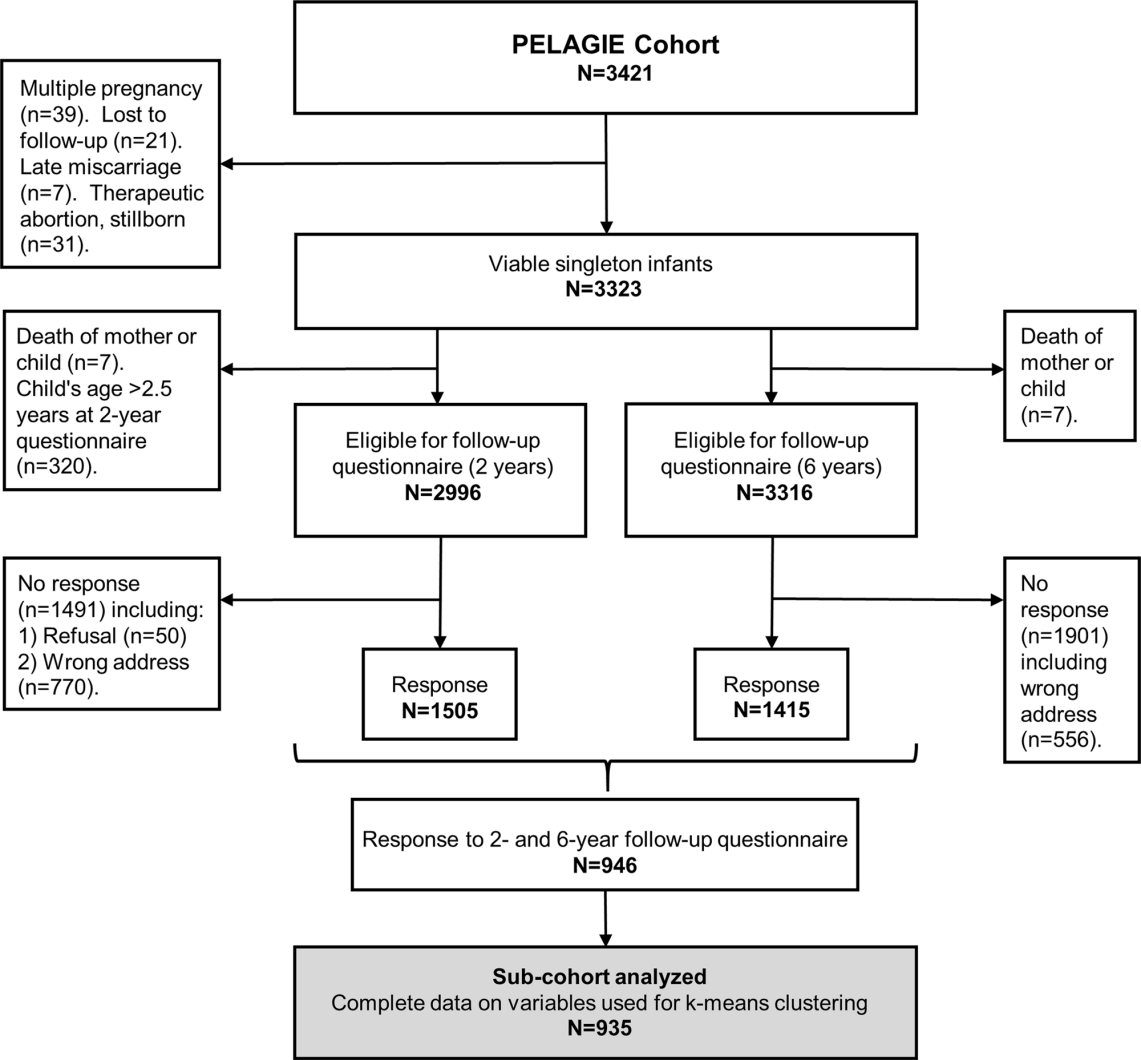
Flow chart of the PELAGIE mother–child cohort. The absence to follow-up at different time points figures on the left (Inclusion, 2-year follow-up) and the right (6-year follow-up) side of the flow chart. Complete data for this analysis were available for 935 mother–child couples

The cluster analysis, included 935 of the 3323 eligible mother–child couples from the PELAGIE (Perturbateurs Endocriniens: Étude Longitudinale sur les Anomalies de la Grossesse, l’Infertilité et l’Enfance) mother–child cohort (Fig. 1).

Compared with the nonrespondents, respondents were older (*p* < 0.001), had higher education level (*p* < 0.001), and were less often nulliparous (*p* = 0.008) or a smoker at inclusion (*p* < 0.001) (Table 1).

**Table 1:** Respondents and nonrespondents to the two-year follow-up questionnaire from the PELAGIE mother–child cohort

Variable	Analyzed subcohort	Nonrespondents	
	*n* = 935	*n* = 2370	
	*N* (%)	N (%)	*p*-Value_1_
*Maternal BMI at inclusion (kg/m²)*_2_			
<18.5	62 (6.6)	181 (7.7)	0.4
18.5 ≤ BMI <25	720 (76.3)	1741 (74.2)	
25 ≤ BMI <30	122 (12.9)	302 (12.9)	
≥30	40 (4.2)	123 (5.2)	
*Maternal age at inclusion (years)*			
<30	423 (44.7)	1269 (53.5)	<0.0001
30 ≤ age < 35	371 (39.2)	790 (33.3)	
≥35	152 (16.1)	311 (13.1)	
*Nulliparous*_3_			
Yes	386 (40.9)	1083 (45.9)	0.008
No	559 (59.2)	1276 (54.1)	
*Maternal level of education*_4_			
Primary or secondary education	124 (13.1)	500 (21.2)	<0.0001
Baccalaureate	161 (17.0)	462 (19.6)	
Higher education	660 (69.8)	1401 (59.1)	
*Maternal smoking at inclusion*_5_			
Non smoker	732 (78.0)	1623 (69.3)	<0.0001
<10 cig/day	142 (15.1)	469 (20.0)	
≥10 cig/day	64 (6.8)	249 (10.6)	
*Maternal alcohol consumption at inclusion*_6_			
Yes	138 (14.7)	345 (14.8)	1.0
No	798 (85.2)	1193 (85.2)	
Gender			
Male	485 (51.3)	1186 (50.0)	0.5
Female	461 (48.7)	1184 (50.0)	
*Cesarean delivery (vs. vaginal)*_*7*_	156 (16.8)	411 (17.8)	0.5
*Preterm birth (* *<* *37 weeks)*_8_			
Yes	30 (28.6)	93 (4.0)	0.3
No	912 (96.1)	2262 (96.0)	
*Low birth weight (* *<* *10% for gestational age)*_9_			
Yes	59 (6.3)	156 (6.6)	0.7
No	885 (93.8)	2212 (93.4)	

*BMI* body mass index (kg/m^2^)

1—chi-square test; Missing data: 2—*n* = 25; 3—*n* = 12; 4—*n* = 8; 5—*n* = 37; 6—*n* = 42; 7—*n* = 73; 8—*n* = 19; 9—*n* = 4

The participating mothers had a mean age of 30.8 years (SD 4.1), and 69.8% had at least some post-secondary education. At inclusion, 22.0% smoked, and 14.7% drank some alcohol. Most of the children were full-term (96.8%) and born by vaginal delivery (83.2%); 51.3% were boys (Table 1). There was a family history of allergy in 249 (26.3%) mother–child pairs. Fewer than half of the children had been exclusively breastfed (41.1%) during the first 3 months. At age 2, 34.8% had been exposed to environmental tobacco smoke (ETS), 44.4% to bleach, 45.0% to glue, and 48.5% to alkyd/acrylic paints used for renovation activities in the dwellings. Half of the children had been exposed to pets at age 2, 40.5% slept on a mattress bought at least 3 years ago, and 41.2% in a bedroom aired less than once a day. Overall, 55.0% of the dwellings used individual heating devices fueled by gas/fuel oil/wood or chimneys (Table 2).

**Table 2:** Characteristics of the analyzed subcohort (*n* = 935)

	Number (%) or mean (SD)
*Maternal characteristics*	
Maternal BMI at inclusion	
<18.5	62 (6.6)
18.5–25	720 (76.3)
25–30	122 (12.9)
≥30	40 (4.2)
Maternal age at inclusion (years)	30.8 (4.1)
Nulliparous (vs. multiparous)	386 (40.9)
Maternal level of education	
Primary or secondary education	124 (13.1)
Baccalaureate	161 (17.0)
Higher education	660 (69.8)
Maternal smoking at inclusion (vs. no)	206 (22.0)
Maternal alcohol consumption at inclusion (vs no)	138 (14.7)
Family history of allergy (yes) _1_	249 (26.3)
*Child’s characteristics*	
Male gender	485 (51.3)
Cesarean delivery (yes)	156 (16.8)
Exclusive breastfeeding during the first 3 months (yes)	389 (41.1)
Preterm birth (<37 weeks)	30 (3.2)
Low birth weight (<10% for gestational age)	59 (6.3)
Height (cm)	49.8 (2.2)
Weight (g)	3419 (484)
*Environmental characteristics*	
ETS at home since birth (yes)	329 (34.8)
Time spent in rooms cleaned with bleach (yes)	420 (44.4)
Use of glues for renovation activities in the first 2 years of life (vs. no)	326 (45.0)
Use of alkyd/acrylic paints for renovation activities in the first 2 years of life (vs. no)	459 (48.5)
Pets at home since birth	472 (50.0)
Child’s mattress ≥ 3 years	383 (40.5)
Ventilation of the child’s bedroom less than on time/day (vs. no)	390 (41.2)
Individual heating using gas/fuel oil/wood/chimneys (vs. no)	520 (55.0)

*SD* standard deviation, *BMI* body mass index, *ETS* environmental tobacco smoke

Missing values: 1—*n* = 137, *n* < 40 for other variables

### Outcome data

**Fig. 2:**
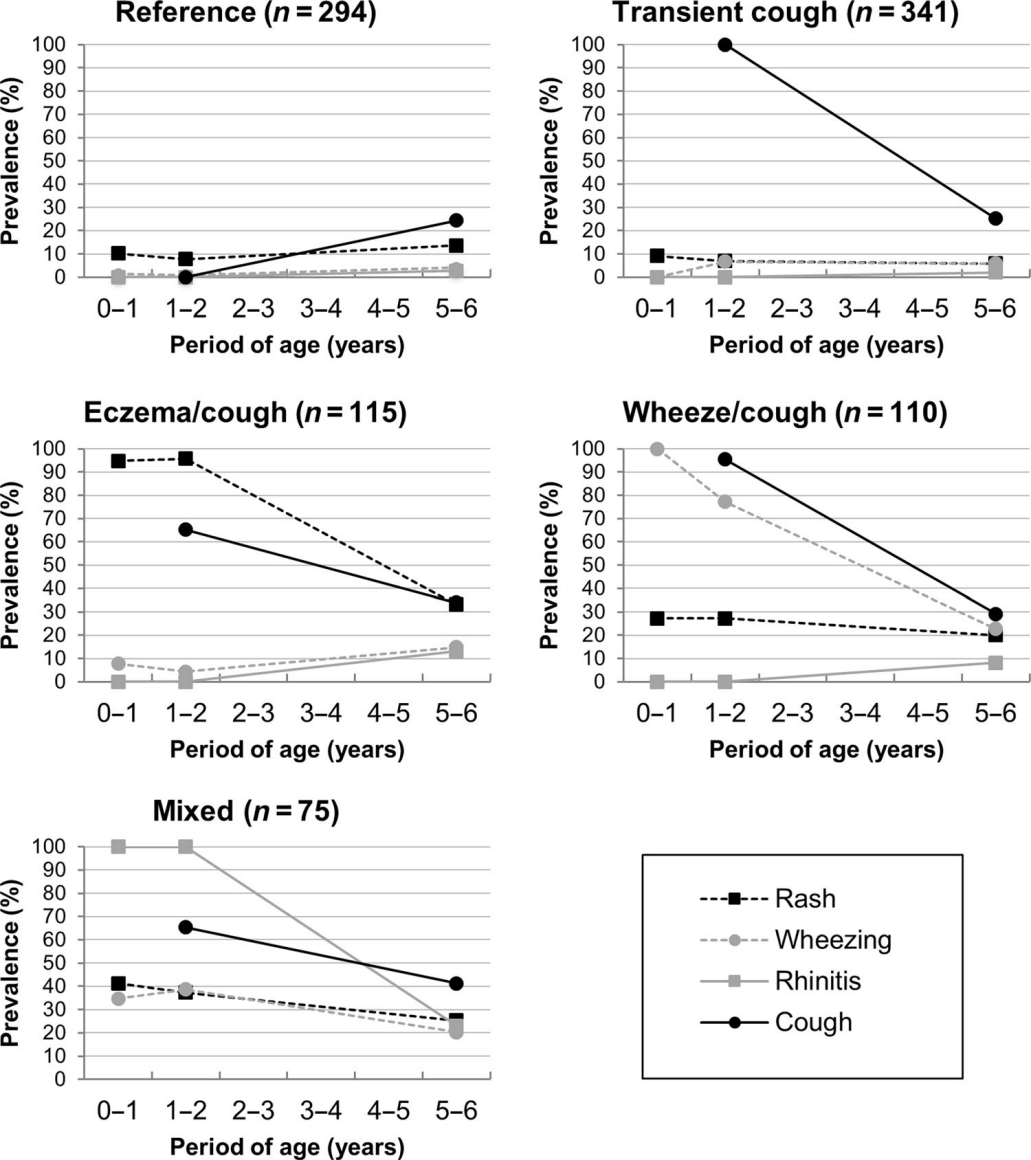
Description of the five-cluster solution obtained in the cluster analysis. Each graph corresponds to one cluster. Asthma and allergy symptoms (rash, wheezing, cough, and rhinitis symptoms) figure as prevalence at each time point (1, 2, and 6 years) and serve to characterize each cluster. The “reference group” had overall low asthma/allergy symptoms

The best partition for the cluster analysis was a five-cluster solution (Fig. 2). The prevalence of respiratory/allergic symptoms (wheeze, rash, rhinitis, and cough) at age 1, 2, and 6 years of the child (except cough, recorded only at age 2 and 6 years) were used to characterize and label each cluster obtained (supplementary Table 1). At time point 1, 4% of the subjects were assigned to a cluster we labeled “reference” phenotype. The prevalence of respiratory/allergic symptoms for this phenotype was quite low and ranged from 0.0 to 13.7%, except for cough, which was prevalent in 24.4% of the children at 6 years of age only. The “transient cough” phenotype (36.4%) had cough in the first 2 years (100%) but had recovered by age 6 to reach 25.2%, almost identical to the “reference” at that age. In the “eczema/cough” phenotype (12.3%), 94.8.% of the children experienced itchy rash in early life, which decreased by age 6 to 33.0%. Cough was more frequent than in the reference group during the first 2 years and was still reported in 34.2% of these children at age 6. The “wheeze/cough” phenotype showed a very substantial decrease in the prevalence of wheezing, from 77.3% at age 2 to 22.7% at age 6. Cough also decreased in this phenotype, from 95.5% at age 2 to 29.1% at age 6. The itchy rash prevalence in this phenotype was 27.3 at age 1 and 2 and 20.0% at age 6. In the “mixed” phenotype, all respiratory and allergic symptoms exceeded prevalence rates from the reference group. Eczema symptoms were more frequent than in any but the eczema/cough phenotype, and wheezing more frequent over time than in any but the wheezing/cough phenotype. Cough at age 6 was more prevalent (42.0%) than in either the wheeze/cough or transient cough phenotype.

The description of other respiratory and allergic health outcomes is available in Supplementary Table 2. At age 2, 64.9 and 72.9% children in the eczema/cough and wheeze/cough phenotypes, respectively, had had otitis media. Most of the children who had ever been awakened by breathlessness belonged to the wheeze/cough phenotype (15.5%), and most of those who had been awakened by respiratory discomfort belonged to the mixed phenotype (41.9%). The mixed phenotype also contained the most children with hay fever (27.0%) and other allergic rhinitis (24.3%). Table 3 summarizes the associations between physician-diagnosed diseases and phenotypes. At the age 6 of the child, 107 (11.4%), 212 (22.6%), and 57 (6.1%) parents declared that a doctor had already diagnosed their child with asthma, eczema, or food allergy, respectively. The highest proportion of the 107 children with physician-diagnosed asthma belonged to the wheeze/cough phenotype (29%). Comparably 29.3% of the 212 children diagnosed with eczema were allocated to the eczema/cough phenotype, but 23.1% were defined to the reference and the transient cough phenotypes. At time point 2, 8 (1%) of children diagnosed with food allergy had been attributed to the eczema/cough phenotype. Logistic regression models showed that the wheeze/cough phenotype was associated only with physician-diagnosed asthma. Both the eczema/cough and mixed phenotypes were associated with all three physician-diagnosed conditions of interest (asthma, eczema, and food allergies). The transient cough phenotype, however, did not differ significantly from the reference group for any of these conditions. Other respiratory and/or allergic symptoms reported by parents are shown in Supplementary Table 2.

**Table 3:** Physician-diagnosed respiratory and allergic diseases in phenotypes

	Asthma_1_		Eczema_2_		Food allergy_3_	
	*N* = 107 (11.4%)		*N* = 212 (22.6%)		*N* = 57 (6.1%)	
	OR (CI 95%)	%	OR (CI 95%)	%	OR (CI 95%)	%
*Phenotypes*						
Reference	ref.	12.2	ref.	23.1	ref.	17.5
Transient cough	1.6 (0.8–3.1)	21.5	0.8 (0.5–1.3)	23.1	0.9 (0.4–2.3)	19.3
Eczema/cough	**4.0 (1.9–8.4)**	16.8	**6.0 (3.7–9.6)**	29.3	**4.5 (2.0–10.3)**	28.1
Wheeze/cough	**8.4 (4.2–16.9)**	29	1.4 (0.8–2.5)	11.8	1.6 (0.6–4.6)	10.5
Mixed	**8.0 (3.7–16.9)**	18.7	**2.2 (1.2–4.0)**	10.9	**5.4 (2.3–13.2)**	21.1

*CI* confidence interval, *OR* odds ratio, *ref* reference

The ORs are calculated in relation to the reference phenotype. Bold aORs differ significantly from the reference (*p* < 0.05). Missing values: 1—*n* = 32: *n* = 83: *n* = 5

### Main results

Factors associated with phenotypes are shown in Table 4. The risk of transient cough and eczema/cough phenotypes were lower in children born to mothers aged 35 years and older and in those with exclusive breastfeeding for 3 months. Family related factors associated with the wheeze/cough phenotype were multiparity, maternal smoking at inclusion, and family history of allergy; the latter was also associated with the mixed phenotype. Children born to mothers aged at least 35 years had a reduced risk of belonging to the wheeze/cough phenotype. An environmental determinant associated with this phenotype was exposure to glues for indoor renovation activities in early life. Children with the wheeze/cough phenotype were at reduced risk at age 2 of sleeping on a mattress bought at least 3 years earlier. The only environmental determinant associated with the mixed phenotype was renovation-related use of alkyd/acrylic paints in early life. The sensitivity analysis (for preterm birth >37 weeks of gestation and birth weight < 10th percentile) did not modify the adjusted model.

**Table 4:** Factors associated with respiratory/allergic phenotypes from birth to age 6 in the PELAGIE cohort

	Phenotypes					*p*-Value
	Reference	Transient cough	Eczema/cough	Wheeze/cough	Mixed	
	*N* = 229	*N* = 238	*N* = 87	*N* = 82	*N* = 58	
		aOR (95% CI)	aOR (95% CI)	aOR (95% CI)	aOR (95% CI)	
*Maternal and family characteristics*						
Maternal BMI before pregnancy ≥ 30 (vs. <30)	ref.	1.8 (0.6–5.0)	2.9 (0.9–9.7)	0.8 (0.2–2.7)	2.4 (0.6–9.0)	0.33
Maternal age at inclusion ≥ 35 years (vs. <35 years)	ref.	**0.5** (**0.3–0.8)**	0.5 (0.3–1.1)	**0.4** (**0.2–0.9)**	0.4 (0.2–1.0)	0.03
Parous (vs. nulliparous) at inclusion	ref.	1.3 (0.9–2.1)	1.0 (0.5–1.8)	**3.1** (**1.6–5.7)**	1.0 (0.5–1.9)	0.01
Maternal smoking at inclusion_1_	ref.	0.9 (0.5–1.6)	0.8 (0.4–1.7)	**2.4** (**1.2–4.9)**	1.1 (0.5–2.5)	0.06
Family history of allergy_1_	ref.	1.4 (0.9–2.3)	1.5 (0.8–2.7)	**2.2** (**1.2–4.0)**	**2.5** (**1.3–4.8)**	0.06
*Child’s characteristics*						
Male gender	ref.	1.2 (0.8–1.8)	1.3 (0.8–2.1)	1.5 (0.9–2.6)	1.2 (0.7–2.1)	0.6
Cesarean delivery (vs. vaginal)	ref.	1.1 (0.7–1.9)	0.6 (0.3–1.4)	1.4 (0.7–2.9)	1.0 (0.4–2.3)	0.6
Exclusive breastfeeding ≥ 3 months_1_	ref.	1.0 (0.7–1.4)	**0.5** (**0.3–0.9)**	1.1 (0.7–2.0)	1.0 (0.6–1.4)	0.1
*Environmental characteristics*						
ETS at home since birth (yes)	ref.	0.9 (0.6–1.4)	0.9 (0.5–1.6)	1.2 (0.7–2.3)	1.3 (0.7–2.6)	0.7
Time spent in rooms cleaned with bleach_1_	ref.	1.1 (0.7–1.6)	1.5 (0.9–2.6)	1.4 (0.8–2.5)	1.5 (0.8–2.7)	0.3
Use of glues for renovation activities (<age 2)_1_	ref.	1.3 (0.8–2.2)	1.0 (0.5–2.0)	**2.3** (**1.2–4.7)**	0.8 (0.4–1.7)	0.1
Use of glycerophtalic/acrylic paints for renovation activities in the first two years of life _1_	ref.	1.1 (0.7–1.7)	1.6 (0.8–3.0)	0.8 (0.3–1.5)	**2.1** (**1.0–4.4)**	0.1
Pets at home since birth	ref.	0.7 (0.5–1.1)	1.0 (0.6–1.7)	0.8 (0.4–1.3)	0.9 (0.5–1.6)	0.5
Child’s mattress ≥3 years old	ref.	0.8 (0.5–1.3)	0.8 (0.5–1.5)	**0.5** (**0.3–0.9)**	1.1 (0.5–2.0)	0.2
Child’s bedroom aerated less than once a day_1_	ref.	1.0 (0.7–1.4)	0.9 (0.5–1.5)	0.9 (0.5–1.7)	0.9 (0.5–1.6)	1.0
Individual heating using gas/fuel oil/wood/chimneys_1_	ref.	0.8 (0.6–1.2)	1.1 (0.7–1.8)	0.9 (0.6–1.6)	0.8 (0.5–1.5)	1.0

*CI* confidence interval, *aOR* adjusted odds ratio

Multinomial logistic regression model [the dependent variable is the five-cluster solution variable obtained in the cluster analysis, (ref = Reference phenotype)]. All the independent variables used in the final model are shown in this table. *R*-square of the full model was 0.14. Bold aORs differ significantly from the reference (*p* < 0.05), 1: vs. no

## Discussion

This study identified five phenotypes of respiratory and allergic symptoms over the first 6 years of life in a population-based mother–child cohort. Phenotypes differed in their physician-diagnosed asthma/allergic diseases and parent-reported comorbidities. We observed an association between some avoidable domestic environmental determinants with respiratory/allergic phenotypes from birth to age 6, specifically, early postnatal exposure to glues and paints.

One of this study’s strengths is its use of an unsupervised statistical method to describe the coevolution of a set of respiratory and allergic symptoms. Unsupervised clustering techniques are particularly appropriate to describe patterns of evolution in a set of variables measured repeatedly like in birth cohorts. KmL3D allows clustering individuals on several trajectory variables jointly and takes into account their possible coevolution while liberating the observer from clinical a priori. Meanwhile, some subjective choices had to be made, basically the number of clusters tested, which was of necessity due to our sample size. We were able to explore symptoms in very early and late preschoolers over a relatively long period and past age 5, the threshold when wheezing starts to be an indicator of asthma. The use of binary data in the *k*-means algorithm might have led to clustering errors in the construction of phenotypes, but these errors are likely to be nonsystematic.

The most important limitation involves the measurement of symptoms on which phenotypes were built. Wheezing was assessed with items derived from ISSAC (The International Study of Asthma and Allergies in Childhood) questionnaire,^3^ validated on 7 year old children. It is uncertain what kinds of respiratory noises parents qualify as wheezing.^13^ The questionnaire is nonetheless widely used in epidemiologic studies in younger children^14^ and was used exclusively in two^4,6^ of the three^4–6^ articles to which we compare this study. Eczema symptoms might be overestimated. Because we did not assess typical localizations of itchy rash, and did not dispose of any eczema-medication data, noneczema symptoms such as xerodermia might have been assessed and could explain a certain degree of misclustering of children with eczema allocated to the reference group and the transient cough phenotype. Different cough measures were used for the follow up of the 2 and 6 year olds. Since we did not dispose of any specific item on cough in the 2 first years of life, we supposed that parents who declared having had a child with bronchiolitis/bronchitis, had had a child with cough. This could be source of misclustering. Data on asthma/allergic symptoms were not collected between ages 2 and 6, so that we lack data about relevant time periods in symptom evolution, in particular, age 3.^15,16^ Correct asthma medication might lead to less symptoms what for our symptom trajectories might be imprecise. But our phenotypes were consistent with most of the physician-diagnosed conditions and other respiratory and allergic symptoms, which reinforces their likelihood. Asthma medication was also consistent with phenotypes since it was more frequent in the wheeze/cough and mixed phenotypes. Misclustering due to under-declared of correctly treated symptoms are unlikely to be differential and would, as the case may be, let the association observed tend towards zero. We, therefore, do not believe that it affected associations observed.

Another limitation is attrition. The questionnaires were self-administered, sent by the postal service, and lengthy (>1 h for completion) due to the broad research scope covered by the cohort. Each of these factors may have discouraged participation and induced bias in association estimates. Because the outcomes measured in this paper do not concern behavioral variables, we believe, in accordance with Greene et al.^17^ that follow-up bias is likely to be small.

Three previous studies have used unsupervised techniques to study multiple longitudinal respiratory- and allergy-related symptoms and to identify phenotypes.^4–6^ Comparison appears thorny; not all studies used only the ISAAC questionnaire,^5^ they considered different variables for phenotype identification, and health outcomes were not measured at the same time points. As Pinart et al.^18^ recently underlined, efforts to standardize the reporting of allergic disease phenotypes in cohorts are needed. However, similarities are apparent, especially for phenotypes. Our wheeze/cough phenotype was exclusively associated with physician-diagnosed asthma and similar to the transient wheeze phenotype identified by Rancière et al.^4^ Likewise, two of the studies^4,5^ identified a group with high levels of eczema symptoms and low levels of other disorders, strongly associated with physician-diagnosed eczema.^4^ Similarities also exist between the persistent sensitization-associated cough/rhinitis phenotype in Rancière et al.^4^ and our mixed phenotype: cough and rhinitis were predominant, moderate wheeze and eczema symptoms remained, and it was associated with all the physician-diagnosed conditions tested (asthma, eczema, and food allergy).

The prenatal and early postnatal period is an important window of vulnerability for developing immune and respiratory systems, which are sensitive to environmental chemicals.^19,20^ Our wheeze/cough phenotype was associated with early-life exposure to glue, and Rancière et al.^4^ observed associations between transient wheezing and postnatal exposure to new particle board furniture. Our mixed phenotype showed associations with early exposure to alkyd/acrylic paints used for interior home redecoration. Glues, particle board furniture, and paints can emit chemical compounds that, inhaled or by direct skin contact, can induce adverse health effects. Early exposure to chemicals used for indoor renovation activity is suspected of harmful effects, including the development of asthma^11,21^ and eczema.^22^ We did not, however, measure these compounds, and their effects are not clearly understood.

The causal relation between postnatal exposure to ETS and respiratory outcomes in children is well established.^9,23^ It was studied in two of the papers cited above^4,6^ and observed by Rancière et al.^6^ in two of their phenotypes, rhinitis and dermatitis. A lack of statistical power due to our smaller sample might explain the absence of association in our study.

Exposure to bleach was not associated with any phenotype. Studies of household cleaning products and their effect on asthma/allergy in children are scarce and conflicting. Maternal exposure to a set of domestic chemicals including bleach has been associated with persistent wheezing in offspring at age 3.^24^ More recent focus on the specific use of bleach during pregnancy has shown no association with wheezing at age 1,^25^ while a cross-sectional study found 10–13-year-old children living in homes cleaned with bleach at least weekly less likely to have asthma, eczema, or sensitization to aeroallergens.^26^ No phenotypes were associated with exposure to pets at home, whose respiratory/allergic health effects on children are currently the subject of debate.^9,27^ Surprisingly, children from our wheeze/cough phenotype were less likely to sleep on a mattress supposed to be laden with house dust mite (HDM) than the reference group. Rancière et al.^4^ observed a positive association between their dermatitis phenotype and the presence of a used mattress (older than 3 years at birth). Reverse causation due to the purchase of a new mattress in symptomatic children explaining our surprising association is improbable because it concerned only 19 children. HDM are thought to be implicated in the development of allergic asthma in children at high risk,^28^ with a nonlinear relation: both very high and very low exposure to HDM are probably protective against asthma and HDM sensitization at age 5.^29^ We treated the age of the mattress as a surrogate for HDM exposure in early life, but we cannot quantify the actual exposure, nor compare our sample to a population at high risk for asthma/allergy.

We do not believe that phenotypes identified represent clinical entities that clinicians should relate to. The development and expression of asthma/allergic disorders depend on gene-environment interactions. From the point of view of the primary care giver, if one focuses on potential prevention targets, to date, genetic predisposition to respiratory/allergic diseases is inaccessible to intervention. This paper aimed to describing respiratory/allergic symptoms in the first 6 years of life. It was quite correctly in line with the currently admitted concept of allergic march. We generated hypotheses on the potentially harmful effect on respiratory and allergic health in children aged 0–6 of some chemical exposures that are directly related to parental behavior (renovation and redecoration) and thus avoidable. If confirmed in observational studies and interventional home-based trials, environmental counseling by primary care givers, because of their close relationship to families and potential knowledge of domestic behavior, could be particularly useful in prevention strategies for respiratory and allergic diseases in childhood.

## Methods

### Study design, participants, and settings

PELAGIE, described previously,^30,31^ is a population-based mother–child cohort that enrolled 3421 women in pregnancy (before 19 weeks’ gestation) in Brittany between 2002 and 2006. Data were collected at birth and when the child was 2 and 6 years old. We studied a subgroup of 935 mother–child pairs for whom respiratory and allergic data were available at both follow ups (Fig. 1). Participants provided written informed consent for data collection, and a national INSERM (Institut National de la Santé Et de la Recherche Médicale) ethics committees approved the study procedures.

### Methods of data collection

Data were collected by a self-administered questionnaire filled out mostly by the mother at inclusion and when the child was aged 2 and 6 years, as well as medical reports at delivery (obstetric data collected by midwives and physical examination of the newborn by a pediatrician).

### Variables

The ISAAC questionnaire^32^ assesses four respiratory/allergic symptoms: wheezing, eczema, rhinitis, and cough. Wheezing was measured at ages 1, 2 (2 years questionnaire), and 6 (6 years questionnaire). At age 1 and 2, wheezing was defined as at least one episode of whistling in the chest or an asthma attack. Children not reported to wheeze but with physician-diagnosed asthma were also considered wheezers. Wheezing or whistling in the child’s chest and/or a wheezy sound there during or after exercise during the last 12 months defined wheezing at age 6. Eczema was defined at ages 1, 2, and 6 by a positive answer to the question: “Has your child ever had an itchy rash which was coming and going in the last twelve months?” Rhinitis symptoms were assessed for the first 2 and the sixth years of life and defined by positive answers to ISAAC questions: “Has your child ever had a problem with sneezing, or a runny, or a blocked nose when he/she DID NOT have a cold or the flu?” and “In the past 12 months, has this nose problem been accompanied by itchy-watery eyes?” The PELAGIE questionnaire did not include any specific item to assess child’s cough at the 2-year follow-up. Cough during the first 2 years of life was defined by a positive answer to the question: “Since birth, has your child ever had bronchitis or bronchiolitis?” At age 6, cough was the occurrence of dry night cough in the past 12 months.

To confirm phenotypes, we assessed parent-reported comorbidities such as otitis media at age 2 or ever awakening due to breathlessness or respiratory discomfort and hay fever or other allergic rhinitis at age 6. They were also described against physician-diagnosed asthma, eczema, and food allergies as at the 6-year follow-up we also asked the parents whether a physician had ever diagnosed those disorders.

We assessed indoor environmental factors known or suspected to be involved with respiratory and allergic symptoms.

At age 2, we assessed: ETS, defined as maternal and/or paternal consumption of at least one cigarette per day at home since birth (yes/no); whether child spends time in rooms cleaned with bleach (yes/no); renovation activities in the dwelling since birth that used glues or either alkyd or acrylic paints (yes/no); usual home heating, defined by individual heating by at least one of the following: gas/fuel oil/wood or chimneys (yes/no).

At age 2, we assessed: pets at home, that is, at least one of the following: cat/dog/rodent/bird/farm animal (yes/no) since birth; daily airing of the child’s bedroom (yes/no); and HDM exposure (yes/no), which was evaluated indirectly by the age of the child’s mattress considered old if purchased at least 3 years earlier.

### Statistical methods

Phenotype identification relied on the joint temporal evolution of wheezing, cough, eczema, and rhinitis symptoms. We used the KmL3D unsupervised clustering method^33^ designed to study the joint longitudinal evolution of several variables over time. This method applies the *k*-means algorithm on longitudinal data (it divides the original sample in clusters of individuals with similar symptom trajectories). Moreover, several outcomes/symptom trajectories are considered simultaneously to compute interindividual distances. Each individual is represented by a matrix with his/her levels of different symptoms (lines) at different time points (columns). The multidimensional Euclidean metrics was used to calculate interindividual distances (Euclidean norm of the distance matrices). As the *k*-means algorithm requires predefining a number of clusters, we ran the *k*-means algorithm 20 times to obtain, respectively 3, 4, and 5 clusters. These numbers of clusters, based on an a priori choice were reasonable regarding our sample size and expected interpretable clinical phenotypes.^4–6^ The choice of the optimal clustering was based on the Davies Bouldin cluster validity index.^34^ For each clustering (different numbers of clusters), a proximity indicator reflecting the average within-cluster distance/between-cluster distance is computed. The optimal clustering minimizes this proximity indicator. The resulting clusters were then interpreted as clinical phenotypes based on the mean symptom trajectories observed in each cluster and our medical expertize. The complete cluster analysis was performed with the R software package KmL3D.^33^

Associations between phenotypes and covariates were estimated with univariate polytomous logistic regression models. We used single random imputation by the mean or median when missing values for covariates did not exceed 5.0%. The final regression model included the lifestyle factors associated with phenotypes in the univariate analysis and all the environmental factors, regardless of the strength of their association with phenotypes. Multivariate regression models were adjusted for maternal body mass index (BMI ≥ 30 kg/m^2^: yes/no) and age at inclusion, parity, maternal smoking habits at inclusion (at least one cigarette per day: yes/no), family history of allergy (mothers’ history of allergy and/or maternal grandparent’s history of asthma: yes/no), child’s gender, vaginal delivery (yes/no), and exclusive breastfeeding for at least 3 months (yes/no). A sensitivity analysis, included preterm children and those with a birth weight <10th centile expected for gestational age. The prevalence of these two characteristics was low and thus not appropriate for use in the adjusted regression model. Logistic regression analyses were performed with SAS 9.4, and results were expressed as odds ratios and their 95% confidence intervals (95% CI). The level of statistical significance was 0.05. Our data were analyzed in 2017.

### Reporting summary

Further information on research design is available in the Nature Research Reporting Summary linked to this article.

## Supplementary information

Reporting Summary

Supplementary Information

## Data Availability

The data from the PELAGIE cohort are not freely available as they are the property of our research institute. Access might be asked by contacting the corresponding author.

## Supplementary information

**Supplementary Information** accompanies the paper on the *npj Primary Care Respiratory Medicine* website (10.1038/s41533-019-0141-y).

## Code availability

Codes used for the cluster analysis can be accessed by contacting the corresponding author.
