# Supplementary material for: Home environment: respiratory and allergic phenotypes from birth to age six in the PELAGIE cohort
Source: NPJ Prim Care Respir Med. 2019 Jul 25;29:29. doi: 10.1038/s41533-019-0141-y (PMC6658488; doi:10.1038/s41533-019-0141-y)
Supplement: Supplementary file 2 — Supplementary Information [file 41533_2019_141_MOESM2_ESM.pdf]

Supplementary table 1. Respiratory/allergic symptoms in phenotypes identified in the clustering-analysis

		Phenotypes									
		Reference N= 294		Transient Cough N=341		Eczema/cough N=115		Wheeze/cough N=110		Mixed N=75	
	Child's age at follow-up (years)	n	%	n	%	n	%	n	%	n	%
Rash	1	30	10.2	31	9.1	109	94.8	30	27.3	31	41.3
	2	23	7.8	24	7.0	110	95.7	30	27.3	28	37.3
	6	40	13.6	20	5.9	38	33.0	22	20.0	19	25.3
Wheezing	1	4	1.4	0	0.0	9	7.8	110	100	26	34.7
	2	3	1.0	23	6.7	5	4.3	85	77.3	29	38.7
	6	12	4.1	20	5.9	17	14.8	25	22.7	15	20.0
Rhinitis	1	0	0.0	0	0.0	0	0.0	0	0.0	75	100
	2	0	0.0	0	0.0	0	0.0	0	0.0	75	100
	6	8	2.72	7	2.1	15	13.1	9	8.2	17	22.7
Cough	1										
	2	0	0.0	341	100	75	65.2	105	95.5	49	65.3
	6	71	24.2	86	25.2	39	33.9	32	29.1	31	41.3

Supplementary table 2: Comorbidities associated with respiratory and allergic phenotypes

	Phenotypes					
	Reference	Transient cough	Eczema/cough	Wheeze/cough	Mixed	
	N=294	N=341	N=115	N=110	N=75	
	%	%	%	%	%	p-value2
Otitis Media 1	58.4	65.0	64.9	72.9	72.6	0.04
Woken by breathlessness	2.7	3.8	7.0	15.5	10.8	<0.0001
Woken by respiratory discomfort	9.2	16.2	13.9	38.2	41.9	<0.0001
Hay fever	4.8	3.5	14.8	10.0	27.0	<0.0001
Other allergic rhinitis	5.8	3.2	15.5	11.9	24.3	<0.0001

Missing values: 1: n=42, else n=2. 2 Chi square test.
